# Meningioma grading via diagnostic imaging: A systematic review and meta-analysis

**DOI:** 10.1007/s00234-024-03404-0

**Published:** 2024-06-21

**Authors:** Tushar Upreti, Sheen Dube, Vibhay Pareek, Namita Sinha, Jai Shankar

**Affiliations:** 1 Max Rady College of Medicine, University of Manitoba, Winnipeg, Canada; 2 Department of Biochemistry, University of Winnipeg, Winnipeg, Canada; 3 Department of Radiology, University of Manitoba, Winnipeg, Canada; 4 Department of Pathology, University of Manitoba, Winnipeg, Canada

**Keywords:** Meningioma, Grading, Diagnostic imaging, Computed tomography, Magnetic resonance imaging

## Abstract

**Purpose:**

Meningioma is the most common intracranial tumor, graded on pathology using WHO criteria to predict tumor course and treatment. However, pathological grading via biopsy may not be possible in cases with poor surgical access due to tumor location. Therefore, our systematic review aims to evaluate whether diagnostic imaging features can differentiate high grade (HG) from low grade (LG) meningiomas as an alternative to pathological grading.

**Methods:**

Three databases were searched for primary studies that either use routine magnetic resonance imaging (MRI) or computed tomography (CT) to assess pathologically WHO-graded meningiomas. Two investigators independently screened and extracted data from included studies.

**Results:**

24 studies met our inclusion criteria with 12 significant (*p* < 0.05) CT and MRI features identified for differentiating HG from LG meningiomas. Cystic changes in the tumor had the highest specificity (93.4%) and irregular tumor-brain interface had the highest positive predictive value (65.0%). Mass effect had the highest sensitivity (81.0%) and negative predictive value (90.7%) of all imaging features. Imaging feature with the highest accuracy for identifying HG disease was irregular tumor-brain interface (79.7%). Irregular tumor-brain interface and heterogenous tumor enhancement had the highest AUC values of 0.788 and 0.703, respectively.

**Conclusion:**

Our systematic review highlight imaging features that can help differentiate HG from LG meningiomas.

## Introduction

Meningioma is the most common type of intracranial tumor and arises from the arachnoid cap cells in the meninges, mostly due to one’s natural aging process [1]. These tumors have been classified into Grades 1, 2, and 3 by the World Health Organization (WHO) based on their histopathological features [2]. Whilst the majority of these tumors are benign (grade 1), they can occasionally be either atypical (grade 2) or malignant (grade 3) type [2, 3]. Higher grade (HG) meningiomas (Grade 2/3) correlate with worse prognosis and require a different management plan than lower grade (LG) meningiomas (grade 1) [4–6]. LG meningiomas may be cured with surgical resection or they may be clinically observed, if asymptomatic. However, HG tumors may require adjunctive radiotherapy alongside surgical resection. Even with adequate treatment, the recurrence rate of meningioma grade 2 is 41% and grade 3 is 75% [7, 8]. Hence why a timely diagnostic distinction between LG and HG meningioma is essential for appropriate treatment planning and improving patient outcomes.

The current gold standard test for grading meningioma is pathological analysis, which requires a tumor sample via biopsy [2, 9]. The tumor sample is then processed by neuropathology and graded based on WHO criteria. However, meningiomas are often located in regions with poor surgical access for biopsy or resection, making tumor grading a challenge [2, 9]. Therefore, there is a need for a non-invasive diagnostic test in these scenarios to effectively plan treatments for meningioma patients.

Many studies have been performed on correlating magnetic resonance imaging (MRI) or computed tomography (CT) features with specific meningioma WHO grades with some features showing significant correlation [10–12]. Although there have been some systematic review and meta-analysis published relating to meningioma, they either limit to correlational analysis or MRI as diagnostic modality [13, 14]. There are a lack of studies validating accuracy of routine CT and MRI features in grading meningioma. We aimed to systematically review all the literature available on the use of routine diagnostic imaging for grading meningioma and conduct meta-analysis to assess the diagnostic accuracy of imaging features for grading meningiomas.

## Methods

Our research question is “what imaging features of routine CT and MRI can accurately differentiate HG from LG meningioma?” The search strategy was developed with the help of our institutional librarian (JL) who has experience with systematic review. The Preferred Reporting Items for Systematic Reviews and Meta-Analyses (PRISMA) 2020 checklist was used to prepare the manuscript. The review protocol was registered with PROSPERO (ID = CRD42021273491).

*Search Strategy-* To answer our research question, a systematic literature search on the topic of “meningioma grading via imaging” in July 2021 and updated in June 2022, was conducted using various keywords, MeSH terms, and alternative term combinations. We used search terms such as “meningioma”, “diagnostic imaging”, “x-ray computed tomography”, “nuclear magnetic resonance imaging”, “cancer grading” and “neuroimaging”. The goal was to achieve the most sensitive search strategy that could later be made specific during our screening phase of the project.

*Inclusion and Exclusion Criteria-* Our review focused on understanding intracranial meningioma in adults (≥ 18-years-old), as the disease is more common in this demographic [1, 3, 4]. Furthermore, meningiomas included in the study had to be classified using WHO grading criteria by pathologists. Imaging modalities included in our study were routine MRI and CT. Routine MRI included T1-weighted, T2-weighted, and diffusion-weighted imaging (DWI) sequences, as well as contrast-enhanced T1-weighted studies. Our study only included qualitative imaging findings and did not include any quantitative analysis. We also omitted studies incorporating artificial intelligence or radiomics. Only primary literature was included in the study, but systematic reviews were screened for any missed primary literature. To be included, studies also had to provide sufficient data for each imaging characteristic to fill out a 2 × 2 contingency table for statistical analysis. Articles were limited to the English language. Studies with less than 10 patients were excluded. Studies with populations from the same hospital during the same time period were also excluded due to the potential bias from redundancy in data.

The search was conducted on MEDLINE, EMBASE, and SCOPUS. The final search result was limited to the English language for MEDLINE. On EMBASE, we again limited all the searches to the English language and excluded all conference abstracts to streamline our search results. For Scopus, final search results were limited to articles, reviews, and the English language.

**Fig. 1:**
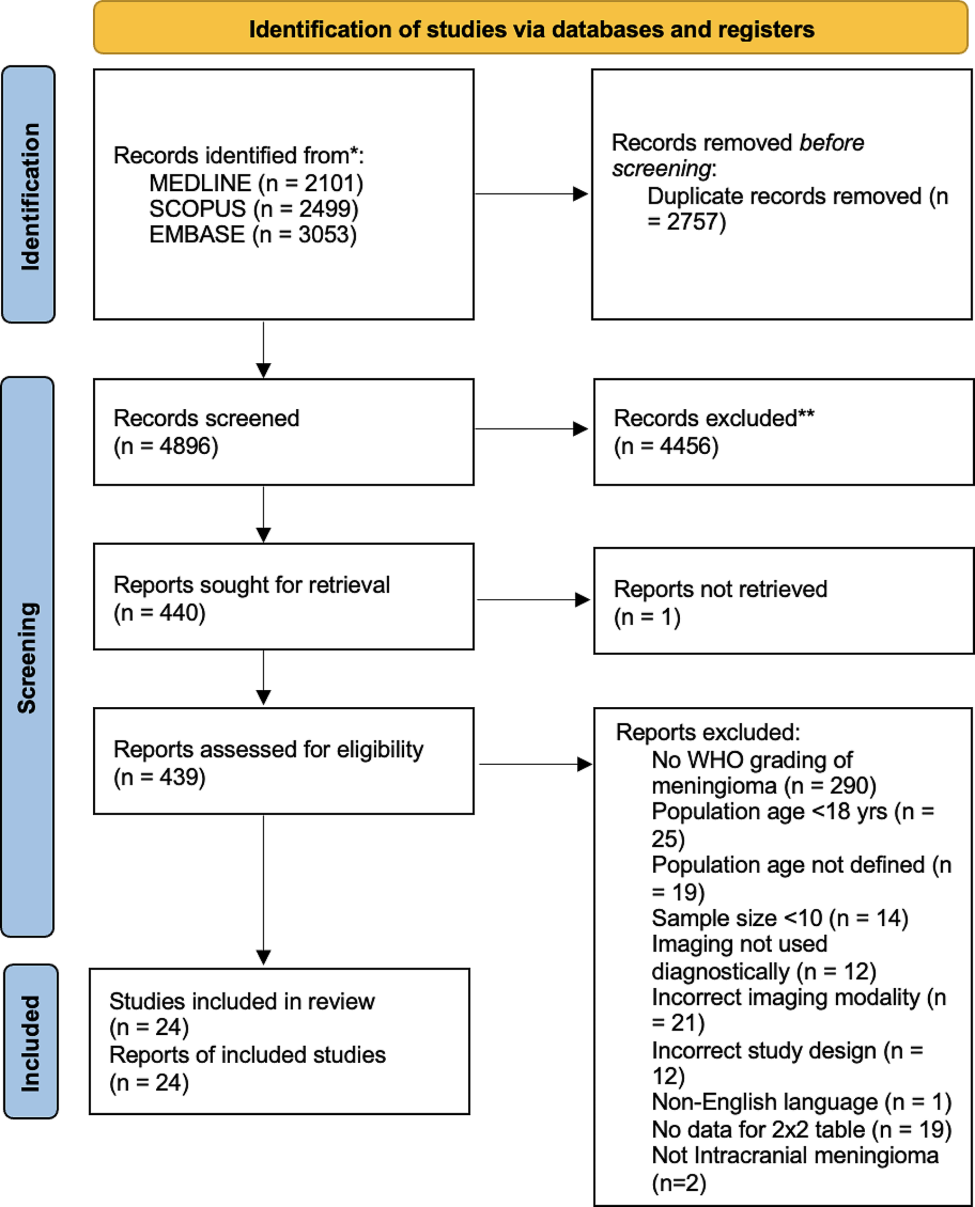
Preferred reporting items for systematic reviews and meta-analyses (PRISMA) flow diagram

*Screening (*Fig. 1*)-* Two independent investigators (TU and SD) were responsible for screening the articles using Covidence. The first screen involved titles and abstracts screening for inclusion/exclusion criteria. The second screening stage involved the full-text revision of relevant articles, which were identified after the title/abstract screening stage. In the end, data was extracted from the set of relevant articles obtained after full-text screening. Conflicts during the screening process were resolved by a team meeting with a senior investigator (JS) and consensus.

*Statistical Analysis-* For each identified imaging characteristic, data were pooled from the included studies to form a 2 × 2 contingency table. Data from meningioma grades 2 and 3 were pooled to form the HG meningioma dataset. Grade 1 meningioma data was designated the LG meningioma dataset. With regards to imaging features, the location of tumors was grouped into either skull base or non-skull base location [1, 4, 15]. The univariate analysis included the calculation of sensitivity (Sn), specificity (Sp), positive predictive value (PPV), negative predictive value (NPV) and area under the curve (AUC) for each imaging characteristic. The chi-squared test was used to evaluate the difference in sex and tumor recurrence between HG and LG meningioma populations. Google sheets were used to tabulate the data. Statistical analysis was conducted using R (R Core Team, 2022) and RStudio (Rstudio Team, 2022).

*Bias and Quality assessment-*In this systematic review, studies included were assessed for quality using the QUADAS-2 tool [16]. The tool helps summarize the review question, construct a flow diagram for the primary study, and judge bias and applicability. Two investigators (TU and VP) reviewed the included articles and extracted key data elements into predesigned data abstraction forms. The expert author was consulted in case of discrepancies in the studies in question or where there was no consensus met among the analyzing authors and the disagreements were resolved by consensus-based discussion.

## Results

After using our search criteria over three different databases and removing duplicate records, we had a total of 4896 studies. After abstract screening, 440 studies were deemed relevant and 439 of those studies were retrieved successfully. 24 studies met our inclusion and exclusion criteria after full text review [10–12, 17–37]. Figure 1 shows the PRISMA flow chart with reasoning behind excluded studies. The most common reason for exclusion of a study was no WHO grading of meningioma. This category included studies that only explored one meningioma grade/subtype and omitted comparing high and low grades of meningioma.

*Characteristic of Included Studies (*Table 1*)-* Out of the 24 included studies, the earliest study was from 2001 and the latest was from 2021. The studies included were from 14 different countries including China, Egypt, England, India, Iran, Italy, Japan, Lebanon, Poland, South Korea, Taiwan, Thailand, Turkey, and USA. China and USA contributed the greatest number of studies with 4 from each country. There were 3 prospective and 21 retrospective cohort studies. 23 studies used MRI of 1.0T, 1.5T or 3.0T and 5 studies used CT as their imaging modality of choice. Only 4 studies included both MRI and CT. The sample size of the included studies varied from 15 to 335 patients. The total number of participants from all included studies was 1909 (586 males and 1323 females). All participants were over the age of 18 with reported mean age ranging from 48.9 years to 64.1 years. Oldest reported participant was 91years old.

**Table 1:** The characteristics of all 24 studies

Author	Study design	Nation	Sample Size	Mean Age in Years (SD)	Sex Sample Size	Imaging Modality
Abdel-Kerim et al. 2018	Retrospective cohort	Egypt	47	Total population: 50.8	M: 10 W: 37	MRI 1.5T
Atalay et al. 2020	Retrospective cohort	Turkey	28	LG population: 64.14 (14.08) HG population: 57.64 (16.32)	M: 9 W: 19	MRI 1.5T
Behzadmeh et al. 2021	Retrospective cohort	Iran	75	LG population: 51.85 (12.18) LG population: 58.86 (17.97)	M: 48 W: 27	CT
Bozdag et al. 2021	Retrospective cohort	Turkey	94	Total population: 51.4 (-)	M: 37 W: 57	MRI 1.5T
Chen et al. 2004	Retrospective cohort	Taiwan	15	Total population: 54 (-)	M: 5 W: 10	MRI 1.5T
Czyz et al. 2017	Prospective cohort	England	54	Total population: 62.4 (14.9)	M: 20 W: 34	MRI, CT
Enokizono et al. 2014	Retrospective cohort	Japan	32	-	M: 8 W: 24	MRI 3.0T
Filippi et al. 2001	Prospective cohort	USA	17	Total population: 55 (-)	M: 4 W: 13	MRI 1.5T
Hale et al. 2018	Retrospective cohort	USA	128	Total population: 55 (-)	M: 34 W: 94	MRI 1.5/3.0T
Hirunpat et al. 2016	Retrospective cohort	Thailand	28	Total population: 58 (-)	M: 9 W: 19	MRI 3.0T
Hsu et al. 2010	Retrospective cohort	Taiwan	75	Total population: 59.3 (-)	M: 20 W: 55	MRI 1.5T, CT
Ilica et al. 2014	Retrospective cohort	USA	65	-	M: 14 W: 51	MRI 1.5/3.0T, CT
Kawahara et al. 2012	Retrospective cohort	Japan	65	LG population: 57.8 HG population: 57.5	M: 26 W: 39	MRI 1.5/3.0T
Lee et al. 2008	Retrospective cohort	South Korea	79	Total population: 55 (12)	M: 19 W: 60	MRI
Lu et al. 2021	Prospective cohort	China	107	Total population: 53.18 (9.86)	M: 24 W: 83	MRI 3.0T
Nowak et al. 2015	Retrospective cohort	Poland	335	Total population: 57.9 (11.3)	M: 89 W: 246	MRI
Rohilla et al. 2018	Retrospective cohort	India	35	-	M: 12 W: 23	MRI 1.5T
Salah et al. 2019	Retrospective cohort	Lebanon	71	LG population: 51.8 (13.8) HG population: 56.92 (14.04)	M: 24 W: 47	MRI 1.5/3.0T, CT
Santelli et al. 2010	Retrospective cohort	Italy	102	Total population: 58.1	M: 28 W: 74	MRI 1.0T
Tan et al. 2015	Retrospective cohort	USA	32	Total population: 60	M: 5 W: 27	MRI 1.5T
Watanabe et al. 2013	Retrospective cohort	Japan	77	-	M: 18 W: 59	MRI 3.0T
Yan et al. 2017	Retrospective cohort	China	131	LG population: 53.00 (8.28) HG population: 52.38 (12.35)	M: 39 W: 92	MRI 1.5T
Yin et al. 2012	Retrospective cohort	China	138	Total population: 54.4	M: 52 W: 86	MRI 3.0T
Yu et al. 2019	Retrospective cohort	China	79	LG population: 48.9 (12.3) HG population: 53.2 (11.3)	M: 32 W: 47	MRI 3.0T

LG – Low grade, HG – High grade, M – Men, W – Women, MRI – Magnetic Resonance Imaging, T – Tesla, CT – Computed Tomography, USA- United States of America

*Imaging Findings (*Table 2*)-* 17 studies reported the sex distribution across HG and LG meningioma. The proportion of males (140/436 = 32.1%) with HG meningioma was significantly (*p* < 0.001) higher than that of females (194/1000 = 19.4%). The recurrence of tumor was reported by 2 studies which showed higher rates of recurrence in HG (24/67 = 35.8%) meningioma compared to LG (27/335 = 8.06%) meningioma (*p* < 0.001).

**Table 2:** The statistical significance of identified imaging features for differentiating high grade from low-grade meningioma

Imaging Feature	Imaging modality	LG population size (%)	HG population size (%)	Odds Ratio	*p*-value
^a^Non-skull base Location [11, 18, 21–25, 29, 30, 34]	CT/MRI	684 (76)	212 (24)	2.18	< 0.001
Mass Effect [11, 18]	CT/MRI	82 (80)	21 (20)	3.85	0.018
Dural Tail Sign [11, 12, 26, 33]	CT/MRI	197 (82)	44 (18)	2.20	0.029
Cystic Change in the Tumor [10, 11, 18, 34]	CT/MRI	183 (72)	72 (28)	5.87	< 0.001
Irregular Tumor-Brain Interface [27, 35]	MRI	149 (69)	68 (31)	14.04	< 0.001
Tumor Capsular Enhancement [21, 27, 35]	MRI	176 (70)	74 (30)	0.26	< 0.001
Heterogenous Tumor Enhancement [11, 12, 17, 20, 21, 27, 33–35, 37]	CT/MRI	449 (71)	187 (29)	6.03	< 0.001
Irregular Tumor Margins [12, 17, 19, 26, 27, 30, 34, 35]	MRI	634 (78)	182 (22)	4.76	< 0.001
Peritumoral Edema [10–12, 17–20, 22, 24, 26, 28–31, 33–36]	CT/MRI	939 (77)	286 (23)	3.24	< 0.001
Tumor Calcification [11, 18, 20, 21, 30, 33, 34]	CT/MRI	460 (78)	128 (22)	2.46	< 0.001
^b^Tumor Hyperintensity on DWI [12, 17, 23, 25, 32]	MRI	157 (76)	49 (24)	5.82	< 0.001
Tumor Necrosis [12, 24]	MRI	138 (70)	59 (30)	6.01	< 0.001
^c^Tumor Hyperintensity on T2-weighted MRI [12, 20, 33, 37]	MRI	118 (70)	51 (30)	0.95	0.886
^d^Tumor Hypointensity on T1-weighted MRI [12, 18, 20, 33, 37]	MRI	132 (67)	64 (33)	1.45	0.229
Reactive Skull Hyperostosis [11, 12, 18, 26]	CT/MRI	182 (77)	53 (23)	0.62	0.161
Skull Invasion [12, 18, 30]	MRI	349 (81)	81 (19)	1.27	0.459

LG = low grade, HG = high grade, SB = skull base, DWI = Diffusion weighted imaging

^a^Reported locations were grouped into either skull base or non-skull base location. With non-skull base having a significant association at differentiating HG from LG meningioma. Tumor location was categorized into skull base and non-skull base using classifications in literature [1, 4, 15].

^b^Hyperintensity of tumor on DWI was pooled into positive predictor, and isointensity and hypointensity were pooled into negative predictor group for 2 × 2 contingency table

^c^Hyperintesity of tumor on T2-weighted MRI was pooled into positive predictor, and isointensity and hypointensity were pooled into negative predictor group for 2 × 2 contingency table

^d^Hypointensity of tumor on T1-weighted MRI was pooled into positive predictor, and isointensity and hyperintensity were pooled into negative predictor group in 2 × 2 contingency table

16 standard CT and MRI features were identified by 2 or more studies for differentiating HG from LG meningioma. These features included non-skull base location of the tumor, mass effect caused by the tumor, heterogenous tumor enhancement, tumor dural tail sign, cystic changes in the tumor, irregular tumor-brain interface, capsular enhancement of the tumor, irregular tumor margins, peritumoral edema, tumor calcification, tumor necrosis, skull invasion by tumor, reactive skull hyperostosis, tumor hyperintensity on DWI, tumor hyperintensity on T2-weighted imaging, and tumor hypointensity on T1-weighted imaging. The presence of these 16 imaging features was associated with HG meningioma. Peritumoral edema was the most investigated imaging feature, identified by 17 studies, and had the largest pooled sample size (939 HG and 286 LG). We found 12 of the 16 features to have a significant association with the meningioma grades (Table 2). Non-skull base location, heterogenous tumor enhancement, cystic changes in the tumor, irregular tumor-brain interface, capsular enhancement of the tumor, irregular tumor margins, peritumoral edema, tumor calcification, restricted diffusion on DWI, and tumor necrosis, all showed statistically significant association with the grade of meningioma. Furthermore, heterogenous tumor enhancement and irregular tumor-brain interface were also determined to have the highest odds ratio of 6.03 and 14.0, respectively. 3 of the 16 imaging features (reactive skull hyperostosis, tumor hyperintensity on T2-weighted MRI and tumor capsular enhancement) had an odds ratio (OR) of less than 1.

The pooled data of each imaging feature was used to determine their diagnostic accuracy, which is outlined in Table 3. We found that the mass effect had the highest Sn and NPV of all imaging features with values of 81.0% and 90.7%, respectively. Cystic changes in the tumor had the highest Sp of 93.4% and irregular tumor-brain interface had the highest PPV of 65.0%. Irregular tumor-brain interface was also the most accurate at identifying HG meningioma at 79.7%. The irregular tumor-brain interface and heterogenous tumor enhancement had the highest AUC values of 0.788 and 0.703, respectively.

**Table 3:** The diagnostic accuracy of significant imaging features for differentiating high-grade from low-grade meningioma

Imaging Feature	Sensitivity (95% CI)	Specificity (95% CI)	PPV (95% CI)	NPV (95% CI)	Accuracy	AUC
Peritumoral Edema*	70.1% [62.2–77.2]	56.1% [51.7–60.3]	31.4% [28.5–34.5]	86.7% [83.6–89.4]	59.2% [55.4–62.9]	0.631
Non-skull base Location*	70.8% [64.1–76.8]	47.4% [43.6–51.2]	29.4% [27.1–31.8]	83.9% [80.7–86.7]	52.9% [49.6–56.2]	0.591
Heterogenous Tumor Enhancement*	61.5% [54.1–68.5]	79.1% [75.0-82.7]	55.02% [49.7–60.2]	83.1% [80.4–85.6]	73.9.1% [70.3–77.3]	0.703
Irregular Tumor Margins*	45.6% [38.2–53.1]	85.0% [82.0-87.7]	46.6% [40.6–52.7]	84.5% [82.6–86.2]	76.2% [73.2–79.1]	0.653
Tumor Calcification*	26.6% [19.2–35.1]	87.2% [83.8–90.1]	36.6% [28.4–45.6]	81.0% [79.3–82.6]	74.0% [70.3–77.5]	0.569
Tumor Hypointensity on T1-weighted MRI	45.3% [32.8–58.3]	63.6% [54.8–71.8]	37.7% [29.8–46.2]	70.6% [65.0-75.6]	57.7% [50.4–64.7]	0.545
Tumor Hyperintensity on T2-weighted MRI	66.7% [52.1–79.2]	32.2% [23.9–41.4]	29.8% [25.2–34.9]	69.1% [58.3–78.1]	42.6% [35.0-50.4]	0.494
Dural Tail Sign*	72.7% [57.2–85.0]	45.2% [38.1–52.4]	22.9% [19.2–27.0]	88.1% [81.7–92.5]	50.2% [43.7–56.7]	0.589
Reactive skull Hyperostosis	26.4% [15.3–40.3]	63.2% [55.7–70.2]	17.3% [11.4–25.4]	74.7% [70.8–78.2]	54.9% [48.3–61.4]	0.448
Cystic Change in the Tumor*	29.2% [19.0–41.0]	93.4% [88.8–96.6]	63.6% [47.6–77.1]	77.0% [74.2–79.6]	75.3% [69.5–80.5]	0.613
Tumor Hyperintensity on DWI*	79.6% [65.7–89.8]	59.9% [51.8–67.6]	38.2% [32.8–44.0]	90.4% [84.2–94.3]	64.6% [57.6–71.1]	0.697
Tumor Capsular Enhancement*	43.2% [31.8–55.3]	25.6% [19.3–32.7]	19.6% [15.7–24.3]	51.7% [43.7–59.6]	30.8% [25.1–36.9]	0.344
Skull Invasion	18.5% [10.8–28.7]	84.8% [80.6–88.4]	22.1% [14.4–32.3]	81.8% [80.0-83.4]	72.3% [67.8–76.5]	0.517
Mass effect*	81.0% [58.1–94.6]	47.6% [36.4–58.9]	28.3% [22.8–34.6]	90.7% [79.7–96.0]	54.4% [44.3–64.2]	0.643
Irregular Tumor-Brain Interface*	76.5% [64.6–85.9]	81.2% [74.0-87.1]	65.0% [56.5–72.7]	88.3% [83.0-92.1]	79.7% [73.8–84.9]	0.788
Tumor Necrosis*	44.1% [31.2–57.6]	88.4% [81.9–93.2]	61.9% [48.6–73.7]	78.7% [74.5–82.4]	75.1% [68.5–81.0]	0.662

PPV = Positive predictive value, NPV = Negative predictive value, CI: Confidence interval, DWI = Diffusion weighted imaging

*imaging feature with *p* < 0.05

**Table 4:** Quality assessment of included studies using the QUADAS-2 tool

Study	RISK OF BIAS				APPLICABILITY CONCERNS		
	PATIENT SELECTION	INDEX TEST	REFERENCE STANDARD	FLOW AND TIMING	PATIENT SELECTION	INDEX TEST	REFERENCE STANDARD
Abdel-Kerim et al.	☹	☺	☹	?	☹	☺	☺
Atalay et al.	☺	☺	☹	?	☺	☺	☺
Behzadmeh et al.	☺	☹	☹	☺	☺	☺	☺
Bozdag et al.	☺	☺	☺	☺	☺	☺	☺
Chen et al.	☹	☺	☹	☺	☹	☺	☺
Czyz et al.	☺	☺	☹	?	☺	☺	☺
Enokizono et al.	☺	☹	☹	☺	☺	☺	☺
Filippi et al.	☺	☺	☺	☺	☺	☺	☺
Hale et al.	☺	☺	☹	☺	☺	☺	☹
Hirunpat et al.	☺	☺	☹	☹	☺	☺	☺
Hsu et al.	☹	☺	☹	☹	☹	☺	☺
Ilica et al.	☺	☺	☹	☺	☺	☺	☺
Kawahara et al.	☹	☺	☹	?	☹	☹	☺
Lee et al.	☺	☹	☹	?	☺	☺	☺
Lu et al.	☺	☺	☹	☺	☺	☺	☺
Nowak et al.	☹	☹	☹	☺	☹	☺	☺
Rohilla et al.	☺	☹	☹	?	☺	☺	☺
Salah et al.	☺	☹	☹	☺	☺	☺	☹
Santelli et al.	☺	☺	☹	☺	☺	☺	☺
Tan et al.	☺	☹	☹	☺	☺	☺	☺
Watanabe et al.	☺	☺	☹	☺	☺	☺	☺
Yan et al.	☺	☺	☹	?	☺	☺	☺
Yin et al.	☹	☺	☹	☺	☹	☺	☺
Yu et al.	☹	☹	☹	☺	☹	☺	☺

☺Low Risk ☹High Risk ? Unclear Risk

The presence of skull invasion had one of the highest accuracies (72.3%) and was highly specific (84.8%) but not sensitive (18.5%) for differentiating the grade of tumor. The presence of hypointensity on T1-weighted MRI had higher diagnostic values in all measures except sensitivity compared to Hyperintensity of T2-weighted MRI (Table 3). Hypointensity on T1-weighted MRI also had the highest AUC value (0.545).

*Risk of Bias -*Table 4 shows the biases in each included study using QUADAS-2 tool. Because all the studies were cohort studies, they inherently have a higher level of bias. Furthermore, there were no control groups included in any of the studies and the blinding of pathologist was present in only 2 studies. Pathologists in all studies used WHO meningioma grading criteria allowing for some standardization of meningioma grading across all studies. Thus, contributing low level of bias in reference standard’s applicability portion of QUADAS-2 tool. The index test’s risk of bias and applicability was comparatively lower than reference standard as 16 of the 24 studies implemented blinding radiologists to the pathologic data.

## Discussion

Our review found meningioma to be more prevalent in females (1323 females to 586 males), similar to what is present in the literature [3]. Regarding the differences in the prevalence of HG meningioma in each sex, we found that HG meningiomas were significantly (*p* < 0.001) more prevalent in males (32.1%) compared to females (19.4%). Furthermore, the recurrence rate was higher in HG meningioma (35.8%) compared to LG (8.06%) meningioma (*p* < 0.001), which is in agreement with the current literature [5, 6]. . The present review included 24 studies evaluating the role of CT and MRI in differentiating the grades of meningioma. 12 CT and MRI features were found to be significant in differentiating HG from LG tumors. Features that had relatively high measures of diagnostic value included irregular tumor-brain interface, heterogeneity of the tumor, mass effect, and cystic changes in the tumor. Irregular tumor-brain interface (AUC = 0.788, OR = 14.0) and heterogenous tumor enhancement (AUC = 0.703, OR = 6.03) were determined to be good diagnostic features for meningioma grading. In addition, the mass effect, had low rates of false positives due to the associated high Sn and NPV values.

Systematic reviews have been conducted in the past that evaluated the potential of diagnostic imaging in prognosticating meningioma and differentiating HG from LG disease. One such study is by Spille et al., where they reviewed primary literature that assessed association of MRI imaging features with either the HG meningioma or the recurrence of meningioma after resection [13]. Spille et al. identified non-skull base location, cystic change of the tumor, irregular tumor-brain interface to be associated with HG disease, similar to our results. They reported calcification to not be significantly correlated with WHO grades, contrary to our results. Whereas bone invasion and tumor intensity on T2-weighted MRI were also not significantly associated with high grade of meningioma, similar to our study. Of note, Spille et al. conducted a systematic review of literature where no meta-analysis was conducted to provide pooled statistics and diagnostic accuracy values for imaging features. Changizi et al. have also systematically reviewed studies to evaluate the role of diagnostic imaging in grading meningioma [14]. They also found peritumoral edema, tumor hyperintensity on DWI, irregular tumor margins, necrosis, dural tail sign, and heterogenous tumor enhancement to be significant (*p* < 0.05) features for differentiating HG from LG meningioma. However, Changizi et al. only evaluated MRI imaging features and studies that had pre-categorized meningioma grades into LG and HG tumors, thus excluding many relevant studies. Because CT continues to be one of the imaging modalities used to assess patients with meningioma routinely, we believed it was important to include it in our study, alongside MRI [1].

The included studies were from North America, Europe, and Asia. This diversity in population is essential for generalizability of the study results to real-world scenarios [38, 39]. Furthermore, the ages included in our study varied from 18-years-old to 91-years-old, although meningioma is most common in the 6th and 7th decade of life [1, 3]. The exclusion of the pediatric population was based on the rarity of the disease in this age group and due to the characteristic differences with higher prevalence of aggressive subtypes [40, 41]. A disproportion of male-to-female ratio was observed. Such disbalance existed due to the higher prevalence of the disease in females when compared to males, thought to be explained by the association of estrogen and progesterone receptors in meningioma [1, 3]. The wide age and geographic distribution included in our study lends external validity to our results [39, 42].

Diagnostic imaging can non-invasively and promptly grade meningioma. Our systematic review and meta-analysis not only outlined all the significant imaging features available for clinically diagnosed meningioma via routine diagnostic imaging, but it also determined the diagnostic test accuracies for 12 CT and MRI imaging features. These results will give clinicians a tool to grade meningiomas that are in surgically challenging locations for biopsy or to avoid biopsy-related risks in many, if not all, patients. However, our study results need to be further validated in a future study to assess if these identified imaging features could help grade meningiomas on imaging. One such study is ongoing in our institution.

### Limitations

All the included studies were cohort studies with the majority being retrospective in nature. Retrospective studies contribute to lower level of evidence due to their inherent biases [43]. Not all studies implemented the blinding of radiologists, whilst only two studies included the blinding of pathologists. This lack of blinding adds further bias to each study result [44]. Due to the lack of high level of evidence available for our research question, our results are impacted by the high risk of bias in included studies and thus require caution while being interpreted. Multivariate analysis could not be performed as not more than 1 study reported the same imaging features.

With regards to the ever-adapting WHO criteria, the grading of tumors has mostly been based on the histopathological analysis, which has not significantly changed over time [2, 45]. However, due to the superiority of molecular profiling in predicting meningioma behavior, molecular profiling has been included in the latest WHO classification [2, 46–48]. As molecular data becomes a routine part of grading meningioma, future studies many need to explore the association of imaging features with the updated WHO grading system that incorporates molecular profiling data.

## Conclusion

In conclusion, we identified 12 features that were significant in differentiating HG from LG meningioma. Features such as mass effect, cystic changes in the tumor, irregular tumor-brain interface, and heterogenous tumor enhancement were significant and had high diagnostic values for predicting HG meningioma. Although the level of evidence from our study is low, our results make clinical impact by outlining the accuracy of features in grading meningioma that radiologists identify routinely on CT and MRI. Consequently, our results will guide future studies in evaluating the use of diagnostic imaging as an adjunct to pathology for grading meningioma by exploring the collective accuracy of significant imaging features that were outlined in this study.
